# Remotely actuated programmable self-folding origami strings using magnetic induction heating

**DOI:** 10.3389/frobt.2024.1443379

**Published:** 2024-08-30

**Authors:** Quentin Lahondes, Shuhei Miyashita

**Affiliations:** ^1^ Automatic Control and Systems Engineering, The University of Sheffield, Sheffield, United Kingdom; ^2^ Insigneo Institute for In Silico Medicine, Sheffield, United Kingdom

**Keywords:** magnetic induction heating, sequential self-folding, origami structures, thermo-responsive, self-folding knot, bio-mimetics

## Abstract

Transforming planar structures into volumetric objects typically requires manual folding processes, akin to origami. However, manual intervention at sub-centimeter scales is impractical. Instead, folding is achieved using volume-changing smart materials that respond to physical or chemical stimuli, be it with direct contact such as hydration, pH, or remotely e.g., light or magnetism. The complexity of small-scale structures often restricts the variety of smart materials used and the number of folding sequences. In this study, we propose a method to sequentially self-fold millimeter scale origami using magnetic induction heating at 150 kHz and 3.2 mT. Additionally, we introduce a method for designing self-folding overhand knots and predicting the folding sequence using the magneto-thermal model we developed. This methodology is demonstrated to sequentially self-fold by optimizing the surface, placement, and geometry of metal workpieces, and is validated through the self-folding of various structures, including a 380 ${mm}^{2}$ croissant, a 321 mm^2^ box, a 447 mm^2^ bio-mimetic Mimosa pudica leaf, and an overhand knot covering 524 mm^2^. Our work shows significant potential for miniature self-folding origami robots owing to the novel sequential folding approach and the ability to achieve remote and tetherless self-folding within constrained environments.

## 1 Introduction

Conventional manufacturing techniques are widely spread for machining centimetric-size objects, but have difficulties reaching smaller ranges for complex mechanisms. In order to produce structures that conform to various metric scales, conventional manufacturing techniques are limited for small structures. To address this limitation, researchers have developed sophisticated systems capable of folding origami structures at the centimeter scale Namiki and Yokosawa (2015); Balkcom and Mason (2004). One successful demonstration involves the folding of a 2-cm origami sheet into a crane using the DaVinci surgical system Ishikawa et al. (2007). Achieving greater precision, Na et al. successfully implemented self-folding techniques where a millimeter-scale octahedron-tetrahedron truss origami with nearly 200 creases folded itself using thermo-responsive smart materials Na et al. (2015).

Smart materials, which can change their volume in response to environmental stimuli, play a major role in the advancements of self-folding techniques Lahondes et al. (2020). When placed between materials that do not change volume, these materials induce a folding motion. Miyashita et al. applied this concept by folding multiple origami robots using a temperature-sensitive shape-memory polymer Miyashita et al. (2017). Shape memory alloys have also been utilized as linear actuators in robots, enabling the compression of foldable structures Kim et al. (2021) and the creation of worm-like robots Onal et al. (2012). Other notable examples include the integration of smart materials into structures through 3D printing of hydrogels Kuang et al. (2019); Uchida and Onoe (2019). Such fine structures are notably valuable in the biomedical field due to their ability to operate in remote and limited-access environments. Applications include wound patching Miyashita et al. (2016), stents Kuribayashi et al. (2006), or drug delivery Denmark et al. (2016); Huang et al. (2019).

Unlike traditional macro-scale methods requiring electrical power input, smart materials can be triggered by changing environmental conditions, including temperature, pH and light, thus enabling direct contact, tethered or remote activation. Direct contact methods utilize pH Zhang et al. (2018); Li et al. (2016), hydration Guan et al. (2005); du Plessis d’Argentré et al. (2018), and temperature-sensitive Iwata et al. (2017); Lahondes and Miyashita (2022) smart materials by immersing the structure in environments with varying characteristics. Tethered energy supplies are commonly used for shape memory alloys Kuribayashi et al. (2006); Zhakypov and Paik (2018), piezoelectric actuators Ma et al. (2013), and other high-power or high-frequency smart materials. To make self-folding structures less dependent on their environment and tethers, self-folding at user-defined timings was achieved relying on remote activation methods including the use of photothermal effects Liu et al. (2017); Li and Liu (2018) or electro-magneto-thermal techniques Ghosh et al. (2009); Liu et al. (2022); Razmjou et al. (2013); Davis et al. (2015). Remote actuation based on magnetic fields can utilize either direct current (DC) or alternating current (AC) magnetic fields. In the case of DC magnetic fields, folding occurs due to the alignment of embedded magnetic particles Tang and Sun (2022); Li et al. (2019) or magnets Iwase and Shimoyama (2006); Boncheva et al. (2005). Conversely, AC magnetic fields induce eddy currents or hysteresis heating, which cause magnetic materials to heat either due to the Joule heating effect or through their constantly changing magnetization with the AC field. When these magnetic particles are embedded in thermo-responsive smart materials, they can bend or fold structures Mohr et al. (2006); Keneth et al. (2023).

Using self-folding techniques, creating complex structures often requires a specific folding sequence to achieve the desired performance. This can be managed by adjusting the hinge sensitivity to specific stimuli Iwase and Shimoyama (2006); Kobayashi et al. (2018), by selective actuation of the hinges Boyvat et al. (2017); Liu et al. (2017), or using multiple smart materials responsive to different stimuli Thérien-Aubin et al. (2013); Downs et al. (2020). Another approach involves separately activating several hinges made of the same smart material Felton et al. (2013); Firouzeh and Paik (2015). Using magnetic fields, this has been demonstrated to fold origami structures by selectively harvesting energy produced by an emitter coil Boyvat et al. (2017); Kening et al. (2011). However, the implementation of electronic components onto miniature structures is limited by size and weight constraints, highlighting the need for low amplitude/frequency magnetic fields to remotely fold origami through the direct heating of bulk workpieces.

**FIGURE 1:**
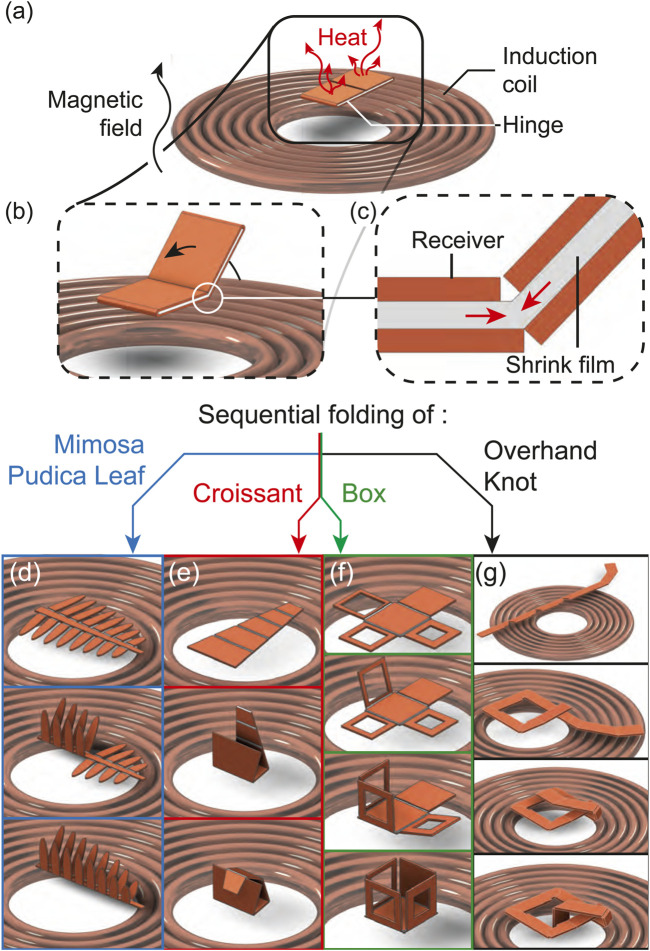
Concept of sequential folding utilizing magnetic induction. Our self-folding structure is placed onto a coil generating an alternating magnetic field which heats up the structure **(A)** and causes it to self-fold **(B)** due to the shrinkage of a film within the structure **(C)**. This enabled the sequential folding of a bio-mimetic leaf **(D)**, a croissant **(E)**, a box **(F)** and an overhand knot **(G)**.

In this study, we used magnetic induction heating, a technique to heat remotely metallic objects using an AC magnetic field (Figure 1A), to self-fold hinges (Figure 1B) by triggering the volume change of a thermo-responsive smart material embedded within the metallic receivers (Figure 1C). The sequential folding of our origami was achieved through the tuning of the sensitivity of the hinges to the heat supplied through a single remote input provided by magnetic induction heating. This was demonstrated for self-folding structures including a bio-inspired Mimosa pudica leaf (Figure 1D), a croissant (Figure 1E), a box (Figure 1F), and a self-folding overhand knot (Figure 1G). The contributions of this article include:

• The development of a method to design self-folding overhand knots and its demonstration.

• The method and demonstration of sequential self-folding of origami through magnetic induction.

• The development of an analytical model to predict the heating rate for induction heating based on the design, material, and layering of the self-folding structure.

• A novel design for in-plane self-folding hinges.

## 2 Methods

### 2.1 Self-folding overhand knot design

Designing string-based structures, such as knots, presents challenges for self-folding techniques such as deformation based on infinite degree of freedom, required folding torque and self-collisions. We propose a methodology to design the knot and position its hinges so that the string can fold with limited degrees of freedom into an overhand knot. Topologically, knots consist of helices with varying pitches, revolutions, and directions, reflecting their chirality.

**FIGURE 2:**
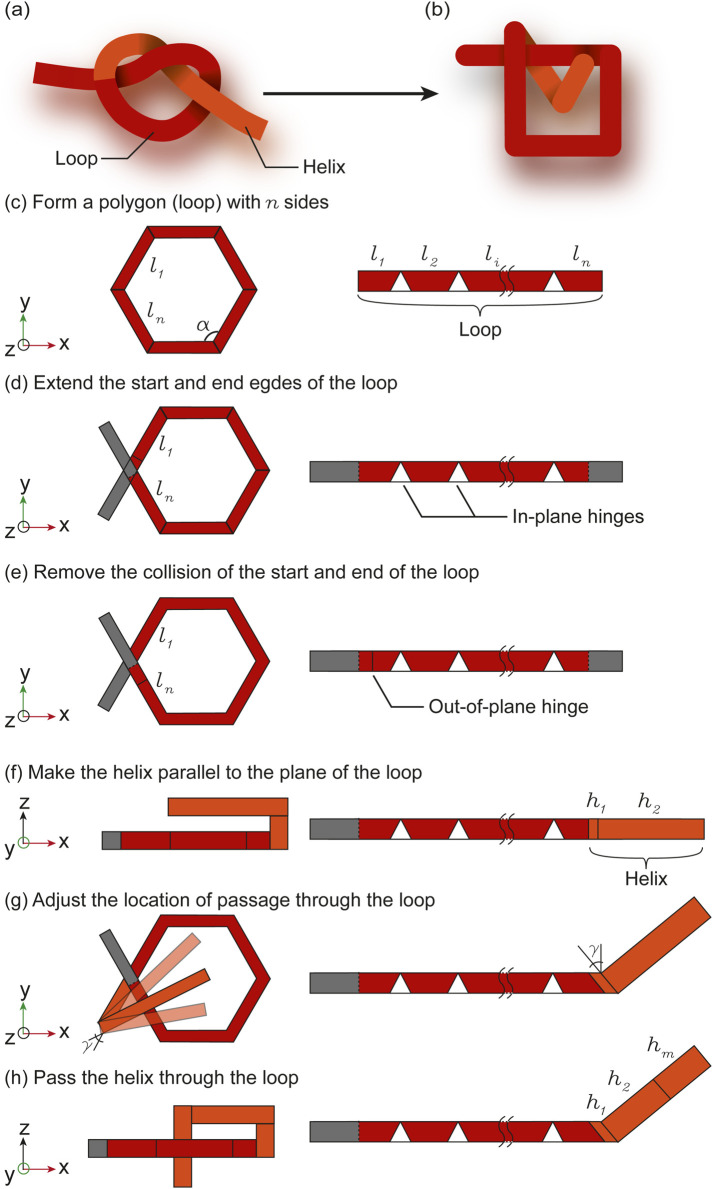
Translation of the overhand knot with continuous deformation **(A)** to discontinuous deformation **(B)**. The loop is formed from a polygon **(C)**, of which a corner is split in two ends that are extended **(D)**. An out-of-plane hinge is placed to avoid collisions between the two ends of the loop **(E)**. The helix is folded parallel to the plane of the loop by placing two out-of-plane hinges on the helix **(F)**. The location of the passage of the helix through the loop is adjusted by tuning the angle γ of the first hinges of the helix **(G)**. The end of the helix is folded through the loop by adding another out-of-plane hinge **(H)**.

To obtain an overhand knot from a folding pattern, we split the knot into a helix and a loop (Figure 2A) which are discretized using hinges (Figure 2B). The loop can be envisioned as any n -sided polygon (Figure 2C), where the first segment $l_{1}$ is the start of the string and the last edge $l_{n}$ connects the loop, and the helix (Figure 2D) made of m segments. The folding pattern consists of n−1 in-plane hinges, represented as triangles and folding in the xy-plane in Figure 2D), and one out-of-plane hinge folding in the xz-plane to prevent collisions between the start and end of the loop as seen in Figure 2E. The first hinge of the helix folds out-of-plane to align the following segment $h_{2}$ parallel to the loop (Figure 2F). This requires two out-of-plane hinges, as a single hinge folding 180° would collide with itself. As illustrated in Figure 2G, the length and orientation of segment $h_{2}$ can be adjusted by orienting the hinges of $h_{1}$ and $h_{2}$ to an angle γ , corresponding to the pitch of the helix, so that the last segment of the helix $h_{m}$ can pass through the loop (Figure 2G). The helix then folds out-of-plane again to pass through the loop (Figure 2H) without colliding with the loop.

**FIGURE 3:**
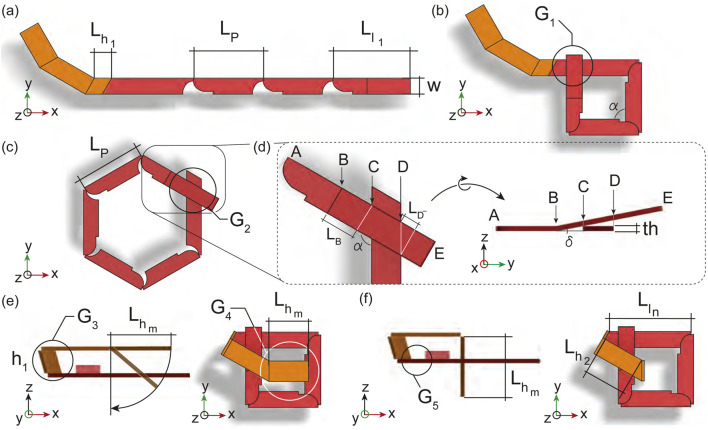
Simulation of the knot folding from the crease pattern proposed by the overhand knot design methodology. The string is initially in its flat state **(A)**, then the loop in red folds **(B, C)** until the ends of the loop crosses **(D)**, the helix places itself parallel to the loop **(E)**, and passes through the loop **(F)**.

With the topology and hinge requirements defined, we ensure the overall geometry of the knot prevents the collisions between the segments during folding. The string has a width w , thickness th , and the segment length L is denoted $L_{li}$ for the loop ( i from 1 to n ) and $L_{hj}$ for the helix ( j from 1 to m ), where i and j identifies the segment (Figure 3A). Each edge of the polygon have the same length $L_{p}$ , except for segments $l_{1}$ and $l_{n}$ , which should extend beyond the loop’s width (Figure 3B) to prevent the first hinge of the helix to be positioned below the loop and for segment $l_{1}$ to prolong the string beyond the loop.

The first geometrical requirement, $G_{1}$ , involves the loop edge length extension. The extension length $l_{D^{-}}$ depends on the polygon considered; given that the segments of the polygon are at an angle α from one another (Figures 3B–D, the lengths of $l_{1}$ can be found as $L_{l1}>L_{p}+L_{D^{-}}$ , where $L_{D^{-}}$ is the minimum length for the segment $l_{1}$ to fully extend beyond segment $l_{n}$ . For any angle below α<90° , $L_{D^{-}}=\frac{w}{\tan\alpha }$ and for α=90° ), the condition changes as $L_{l1}>L_{p}+w$ since $L_{D^{-}}=0$ . The second geometric constraint $G_{2}$ , Figure 3C, concerns the placement of the out-of-plane hinge of $l_{1}$ , which should be at a minimum distance from the segment $l_{n}$ to fold and avoid the collision of $l_{1}$ and $l_{n}$ . The exact length $L_{B}$ can be calculated from the thickness of the string and its folding angle δ as $L_{B}=\frac{th}{\sin\delta }$ in Figure 3D. Figure 3E shows the geometrical constraints $G_{3}$ that ensures the parallelism between segment $h_{2}$ and the loop’s plane. $L_{h1}$ should be at least twice the string thickness to prevent collision of the helix hinge $L_{h1}$ with the loop segments (Figure 3C). $G_{4}$ pertains to the passage of segment $L_{hm}$ through the loop (Figure 3E). During folding, the projected length of segment $L_{hm}$ in the loop plane should not extend beyond the loop internal edges to avoid collisions. Additionally, segment $h_{2}$ must be short enough to position segment $h_{m}$ while preventing its collision with the loop during folding. The fifth geometrical requirement $G_{5}$ pertains to the length extension of $l_{n}$ beyond $l_{1}$ which ensures that the first hinge of the helix is not under $l_{1}$ which requires its placement at a minimum distance of $L_{P}$ from the start of segment $l_{n}$ as seen in Figures 3D, F. Using this methodology, a simulation of the folding of the overhand knot was made to validate its kinematic and geometry as seen in Supplementary Video S1.

### 2.2 Design of self-folding in-plane hinges

**FIGURE 4:**
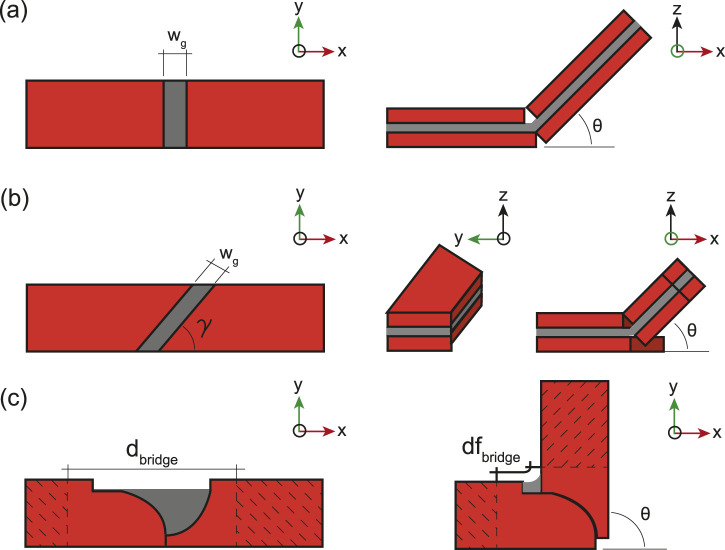
Types of self-folding hinges used in this study. Straight out-of-plane hinge **(A)**, angled out-of-plane hinge **(B)**, and the newly developed in-plane hinge **(C)**.

To create the in-plane hinges necessary for the loops shown in Figures 2, 3, placing an out-of-plane hinge on its side (Figure 4A) is possible, although when using magnetic induction heating, maximizing the surface area of induction, that is the surface of the receiver perpendicular to the magnetic field direction, is critical. Therefore, a new hinge design maintaining the surface area of induction, regardless of the folding angle, and limiting the required folding torque to ensure scalability is essential. To address these requirements, we developed a hinge design that remains in-plane while folding (Figure 4C).

As illustrated in Figure 4C, the in-plane hinge consists of two round inductor receivers made of copper (in orange) interconnected by a polyvinyl chloride (PVC) shrink film smart material (in grey) which, upon heating above 65° C, contracts and pulls the copper receivers together. For effective folding, the bridge length between the induction receivers must exceed the maximum shrinkage of the bridge after heating, denoted as $df_{\mathrm{bridge}}$ . This condition is constrained by ϵ , the shrinkage extent of the smart material, expressed as $di_{\mathrm{bridge}}\epsilon <df_{\mathrm{bridge}}$ . To optimize the performance of the hinge, the PVC film is partially glued to the induction receivers, allowing for a greater length of film to shrink (Figure 4C). Additionally, the top and bottom layers are made of a single piece which is folded onto itself, preventing the PVC film to escape from between the receivers and thus, preserving the bridge length design. Using this design, in-plane hinges could be fabricated and self-folded as seen in Supplementary Video S2.

### 2.3 Fabrication process and experimental setup

The materials used include a 50μ m-thick copper sheet from MacMaster-Carr (9053K532) serving as induction receivers, a 24μ m thick PVC film from SupVox (L119Y80C162AM110OBS7) able to shrink 40% in its length and 20% in its width when exposed to temperatures exceeding its glass transition temperature of 65° C, a 127μ m thick Dura-lar sheet used to rigidify the self-folding structure while rendering it visible in the infrared spectrum for temperature imaging realised by the thermal camera Flir C3-X. Additionally, a double-sided silicone tape of thickness 25μ m from MacMaster-Carr (7615A619) serves as a bonding layer to interface the many layers of our self-folding structure. To heat up the induction receivers, the induction coil was designed to generate an alternative magnetic field on a 60 cm^2^ circular area oscillating at 140 kHz and of 3.2 mT amplitude. More information about the induction setup as well as the fabrication of the out-of-plane and in-plane hinges can be found in Supplementary Sections S1, S2.

### 2.4 Layer configurations

Miniaturizing self-folding hinges for magnetic induction-based folding is challenging, as reducing the structure’s surface area of induction decreases the power induced in the copper receivers. This subsection proposes a receiver layer configuration to enhance the power reception from the induction coil enabling smaller self-foldable structures, and introducing sequential folding by tuning the heat rates between induction receiver.

**FIGURE 5:**
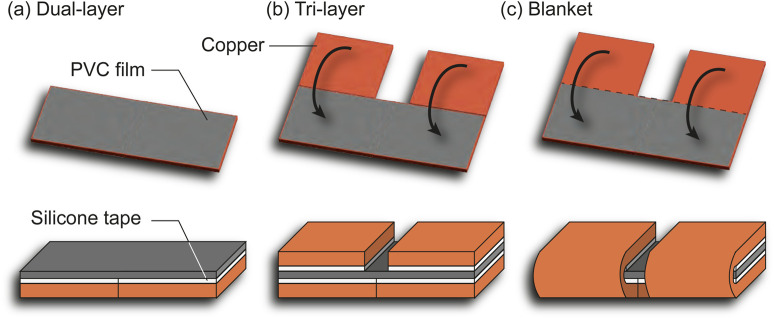
Layer configuration including the dual-layer **(A)**, the tri-layer **(B)** and the blanket configuration **(C)**.

The common dual-layer structure (Figure 5A) allows heat loss from receivers to the environment and the smart material. A conventional alternative is the tri-layer configuration (Figure 5B), which sandwiches the smart material between passive rigid layers known as structural layer. This setup traps the heat and doubles the receiver surface area of induction, increasing the heat rate. Additionally, the structural layers in the tri-layer configuration act as mechanical limiters to regulate the folding angle. Our approach, the blanket configuration (Figure 5C) interconnects top and bottom receivers and simplifies the fabrication process by manually folding the copper receivers around the smart material, instead of manually positioning the top copper receivers onto the PVC film.

### 2.5 Model of the magnetic induction heating

The purpose of the model is to predict the folding sequence by considering factors such as the heat rates of the copper receivers, their design, materials, position on the coil, and magnetic field characteristics.

Induction heating leverages oscillating magnetic fields that induce eddy currents in materials. These induced currents flow along the perimeter of the receiver, generating heat through the Joule heating effect. Furthermore, the electrons moving within the copper receivers are affected by the skin effect, which causes the current to concentrate near the edge of the receivers. This phenomenon ensures that higher frequencies result in shallower skin depths (the depth of the current concentration), thereby localizing the heating effect to the perimeter of the receivers.

**FIGURE 6:**
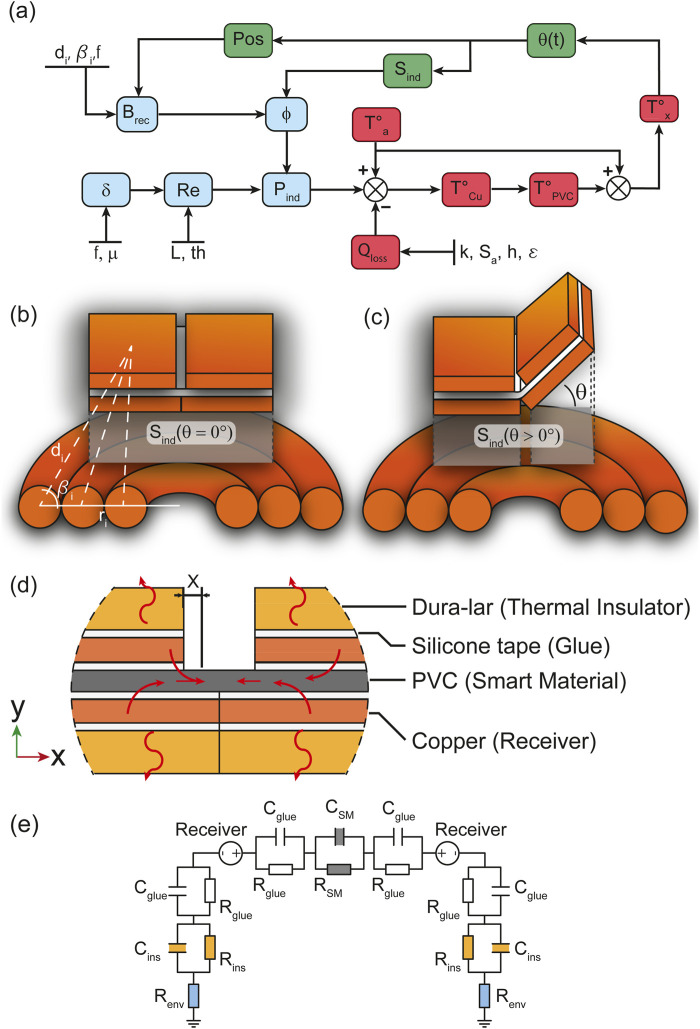
Overview of the model **(A)** showing how electro-magnetic, thermal and mechanics interact to describe the self-folding hinges. The coupling variation between receiver and coil when the hinge’s receiver is flat **(B)** and folding **(C)**. The sideview of the multi-layer self-folding structure showcasing the structure layout and thermal exchange within the it **(D)**. The electric equivalent of the heat transfer within the multi-layer structure illustrating heat storage and conductivity **(E)**.

We divided the model into three components: magnetic, thermal, and mechanical, depicted in Figure 6A in blue, red, and green, respectively. The electro-magnetic analysis calculates the power induced in the receiver $\left(P_{\mathrm{ind}}\right)$ based on the amplitude of the magnetic flux density received $\left(B_{\mathrm{rec}}\right)$ , its frequency (f) , the relative permeability of the receiver $\left(\mu_{r}\right)$ , the folding angle (θ) , and the initial distance $\left(d_{i}\right)$ between the induction coil wires, noted with index i , and the induction receivers. The thermal analysis calculates the temperature of the copper receivers $\left(T_{Cu}\right)$ by accounting for heat loss $\left(Q_{\mathrm{loss}}\right)$ within the structure and the power induced in the copper receivers. Lastly, the mechanical analysis connects the heat received by the PVC film to the folding angle and position of the hinge in space (Pos) , providing information on the position of the structure and the new surface area of induction of the copper receiver.

#### 2.5.1 Power induced and receiver design

The primary objective of the magnetic section is to determine the electrical power delivered to the receiver. Initially, the magnetic field received $B_{\mathrm{rec}}$ is calculated as a function of the receiver’s position on the coil using the Biot-Savart law (Equation 1):

$$B_{\mathrm{rec}}=\overset{n}{\underset{i=1}{\sum }}\frac{\mu }{4\pi }\frac{I\;2\pi r_{i}\;\sin\left(\beta_{i}\right)}{d_{i}^{2}},$$ (1)

where the index i identifying the n wires of the coil, $\beta_{i}$ is the angle between the induction receiver and the wire, $r_{i}$ is the radius of wire i relative to the coil’s center, I is the current through the coil, and μ is the magnetic permeability. To simplify calculations, we assume a uniform magnetic field along the induction receiver at a given position during folding, with $d_{i}$ calculated from the induction receiver’s center of mass above the coil. While this assumption simplifies the model, it limits its accuracy for long induction receivers. If an infinitely long induction receiver were placed on the coil, only its portion on the coil would heat up, but the model predicts a temperature increase on its entire surface.

**FIGURE 7:**
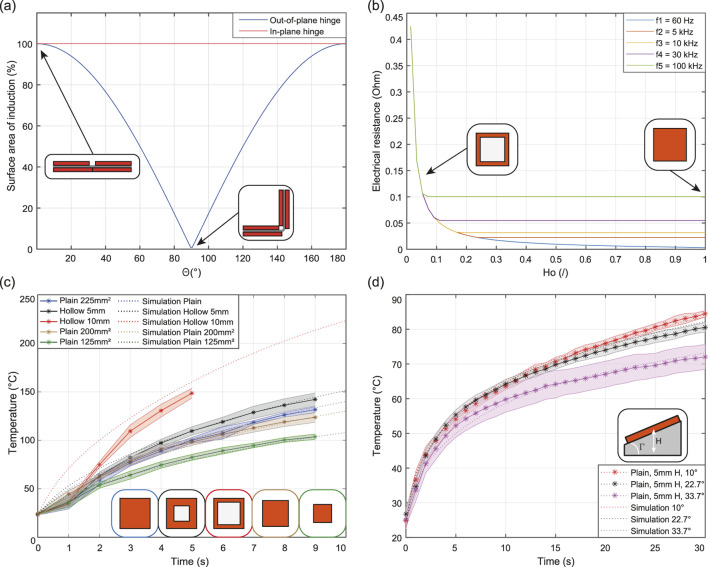
Results of the modelling and experimental characterisation. The surface area of induction against the folding angle for the in-plane and out-of-plane hinges **(A)**. Variation of electrical resistance as a function of the hollowness ratio of a 15×15×0.05 mm^3^ receiver **(B)**. Modelling and experimental characterisation of the temperature rise with receivers with different surfaces and hollowness **(C)**. Model and experimental data highlighting the temperature rise of receivers and their decoupling as the angle between the induction and receiver is increased **(D)**.

From the calculation of the magnetic field received (Equation 1), the power induced within the induction receivers can be calculated as shown in Equation 2:

$$P_{\mathrm{ind}}=\frac{-\frac{d}{dt}{\left(B_{\mathrm{rec}}\;\;S_{\mathrm{ind}}\left(\theta \right)\;\cos\theta \right)}^{2}}{R_{e}},$$ (2)

where $S_{\mathrm{ind}}\left(\theta \right)$ is the surface area of induction corresponding to the projection of the receiver’s surface onto the plane of the coil as shown in Figures 6B, C, $R_{e}$ is the electrical resistance of the receiver. According to this equation, power generation depends on the folding angle θ , resulting in zero power generation when the folding angle approaches 90° , thus illustrating the power generation capability between in-plane and out-of-plane hinges as depicted in Figure 7A. This 90° angle illustrates how a structure that self-folds out-of-plane slowly decouples from the induction coil. It causes the heat generated to gradually decrease until the heat loss in the system balances out the heat generation, leading to a stalling region around 90° specific to the power generation. The surface area of induction where the power generation is sufficient to self-fold the structure is denominated as the self-folding region, which is influenced by the receiver material, design, distance from the center of the coil, shrink film glass transition temperature, layer configuration and the induction coil.

Another factor is the electrical resistance $R_{e}$ as it Introduced in Equation 2, the electrical resistance Re depicts the geometrical dependencies reflecting the design of the copper receiver as demonstrated below in Equation 3:

$$R_{e}=\rho_{e}\frac{C_{o}}{S_{e}},$$ (3)

with $\rho_{e}$ being the electrical resistivity of the receiver, $S_{e}$ the section of the receiver conducting the current, and $C_{o}$ the path length followed by the current, assumed here to be the contour of the receiver of which, a depth δ from the contour towards the inside the of conductor is used by the electrons (Equation 4):

$$\delta =\sqrt{\frac{2\rho_{e}}{2\pi f\mu }},$$ (4)

Consequently, as electrons generate heat via the Joule heating effect, the sides of the receiver heat more than its center, causing the central part to function as a heat sink. This suggests that removing the central material can increase the heat rate of receivers and the folding rate. This is validated by the plot in Figure 7B, which shows the change in the resistance of an induction receiver as a function of the hollowness Ho , defined as the ratio of the receiver’s metal surface to the surface area of induction $S_{\mathrm{ind}}$ . For Ho=1 , the surface is plain (no holes), and as Ho decreases, the hole size increases. The graph indicates that as long as the hole size does not infringe on the conducting width δ , the electrical resistance remains unchanged, implying that any change in the heat rate between a plain and hollow conductor is purely a thermal effect.

#### 2.5.2 Heat generation and losses

This section examines the thermal characteristics of the system to determine the start of the self-folding, which occurs when the copper receiver reaches 65° C or, in other words, starts decoupling from the coil and heat generation diminishes.

Figure 6D; Supplementary Figure S1i in the SupplementaryMaterial illustrate the PVC film positioned between layers of silicone tape, copper receivers, and Dura-Lar thermal insulators. The heat generated by the copper receivers is transferred through these layers, characterized by their thermal conductivity (k) and heat capacity $\left(C_{p}\right)$ which can be interpreted as the resistance to temperature changes and the storage of heat. From this, an electrical analogy is drawn in Figure 6E representing the structure’s layer as a series of capacitors (C) and resistors (R) for each material, except for the induction receiver, which additionally acts as a heat source. Optimising the system for sequential folding consists of differentiating the overall resistance and/or capacitance towards the environment or the PVC film along the structure; increasing the heat transfer can be achieved by reducing the materials thickness and their mass or changing to a more thermally conductive material. More details about our materials thermal properties can be found in Supplementary Section S3. Based on Figures 6D, E, the block diagram thermal model of the system was built (Supplementary Figure S4 using Simulink, where the temperature of the thermal insulator is predicted as a function of the thermal properties of each layer of the structure and the electro-magnetic input from the coil system calculated by Matlab, From experiment to experiment, the geometry of the receivers along with the heat loss were adjusted to fit an experimental curve and the values were kept constant for the rest of that modelling. Notably, the convection coefficient h was adjusted between $20\;W/{}^{\circ}K\;m^{2}$ for the modelling with hollow induction receiver and $46\;W/{}^{\circ}K\;m^{2}$ for the modelling of inclined induction receivers.

The heat trapped between the receivers can only be conducted towards the hinge, where the PVC film is free to shrink. According to the folding angle prediction model Tolley et al. (2014), the PVC film needs to shrink by 10.3% to achieve complete folding, regardless of the hinge gap, $w_{g}$ , shown in Figure 4, defined as the distance between the top receivers. Given that the PVC film used in this study shrinks to 60% of its initial length, 25% of the PVC film’s shrinkage potential should be activated by the copper receivers to achieve complete folding. Thus, the length of PVC film, x , in the hinge gap, illustrated in Figure 6D, that should reach 65° C can be predicted, the temperature along the PVC film, $T_{x}$ , calculated, and the folding angle estimated. More information can be found in the Supplementary Section S3.

For simplicity and visibility in infrared, the following characterization was performed on a receiver/adhesive paper bilayer. The heat conduction from the periphery towards the center of the receiver was neglected, as the measured point was at the periphery due to the skin effect. Despite potential inaccuracies in folding sequence predictions, this assumption is relevant since the self-folding hinges are located at the corners of the receivers.

The first thermal simulation demonstrates the design’s impact on the heat rate by varying the hollowness of square copper receivers. Copper receivers with surfaces of 225, 200, and 125 mm^2^ were tested, along with hollow receivers with Ho=0.89 (5 mm hole) and Ho=0.55 (10 mm hole), covering surfaces of 200 and 125 mm^2^, respectively. By changing the mass of the copper receivers and its surface for the conduction transfer in the simulink model, the block diagram can be adjusted for each geometry. To validate the results of the model, five trials were conducted for each case, and the temperature rise over time is plotted in Figure 7C. The experiment lasted 9 s, except for the 10 mm hollow sample, which was stopped at 5 s due to the fumes production likely from the adhesive of the paper. The hollow receiver with a 5 mm hole heated 10° C and 20° C more than the induction receiver with equivalent surface area of induction and surface covered respectively and similarly, the hollow receiver with a 10 mm hole heated 50° C and 78° C more than its plain counterparts.

The Simulink thermal model accurately followed the experimental trends with a discrepancy of less than 3° C for all curves except for the 125 mm^2^ receiver, which showed a 12° C difference accentuating as the experiment continues. The curves were obtained with the convection coefficient, h , adjusted to match the 125 mm^2^ plain receiver curve and kept constant for other curves. More information on the physical characteristics and model structure is available in the Supplementary Figures S4, S5. In conclusion, hollow receivers exhibit higher heat rates than plain ones, confirming that removing the central material increases heat generation. However, the reduced specific heat capacity may enlarge the stalling region due to the decreased heat storage.

#### 2.5.3 Self-folding and stalling angle

This section analyzes the impact of copper receiver positioning on heat generation and folding behavior. In the second simulation, a 225 mm^2^ receiver was inclined at an angle Γ of 10° , 22.7° , and 33.7° around an axis 12 mm above the induction coil center. Adjustments to the convection coefficient (h) , and heat losses towards the tilting block were introduce thus, maintaining a discrepancy below 2° C in final temperatures. Figure 7D shows that increasing the coil/receiver angle reduces the final temperature from 83.5° C for 10° slope to 73° C for 33.7° slope after 30 s. This experiment illustrates how increasing the coil/receiver angle results in gradual decoupling, reducing the heat generation and validating the assumption that, at this scale, the variation of the magnetic field along the receiver can be neglected.

As the receiver folds towards 90° , decoupling occurs between the coil and the copper receivers, thus slowing the folding speed until stalling. This angle depends on the smart material’s shrinkage temperature; the higher the activation temperature, the more heat is required, and the sooner the hinge enters the stalling region. Conduction losses and heat capacity also influence the stalling angle by affecting the receiver’s heat rate. Heat circulation through layers creates a delay between the PVC film shrinkage and the start of induction heating, providing a time window to supply enough heat to fold past the stalling region. However, this delay may impede the hinge function by hindering the heat dispersion. Precautions against overheating are necessary since PVC melts at 136° C and releases a corrosive gas (Hydrogen Chloride) at 135° C, hence air extraction is recommended to utilize similar structures.

## 3 Results

This section presents the implementation of the design and model conclusions to achieve sequential folding through magnetic induction. The process is demonstrated by self-folding various structures, including a bio-inspired Mimosa pudica leaf, a croissant, a box, and an overhand knot.

### 3.1 Single hinge self-folding

**FIGURE 8:**
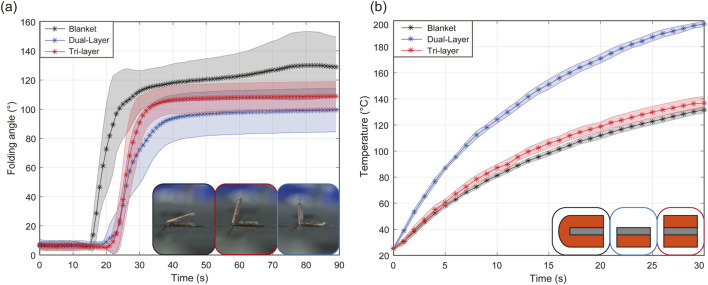
Experimental data of the folding angle of hinges **(A)** and temperature increase **(B)** depending on the layer configuration.

To assess the heat generation as a function of the layer configuration, six dual-layer, seven tri-layer, and eight blanket configurations, each with 5 mm wide square receivers and complemented with 127μ m-thick Dura-Lar layer, were tested. The Dura-Lar layer limited their folding angle to a maximum of 160° . As shown in Figure 8A and Supplementary Video S3, the blanket configuration initiated folding 15 s earlier than the tri-layer and dual-layer. Initially folding at rates of 6.5% /s, 3.6% /s, and 2.7% /s of the target angle for the blanket, tri-layer, and dual-layer configurations respectively, their speeds reduced to 0.16% /s, 0.053% /s, and 0.059% /s after reaching the stalling angle at 90° . The blanket hinge demonstrated higher folding speeds and angles, passing the stalling angle in 71% of trials, indicating better resilience to stalling compared to tri-layer and dual-layer configurations. However, the angle deviation reached 21° compared to 11° and 15° for the tri-layer and dual-layer, respectively. These experimental deviations can be attributed to misalignment during the hinge manufacturing and variations in electrical resistance due to the compression of the blanket’s top/bottom layer connection during fabrication. However, in Figure 8B, the temperature increase of the configurations show that the dual-layer heats at a much higher rate than the tri-layer and blanket configurations. The dual-layer exhibits significant heat loss, while the tri-layer and blanket configurations generate, retain and transfer heat more effectively due to their additional surface of induction and trapping of the PVC film between heating receivers, thereby reducing the heat loss to the environment. Additionally, the electrical connection between the top and bottom receivers in the blanket configuration doubles the electrical resistance, thereby increasing the power induced.

### 3.2 Receiver size for sequential folding

**FIGURE 9:**
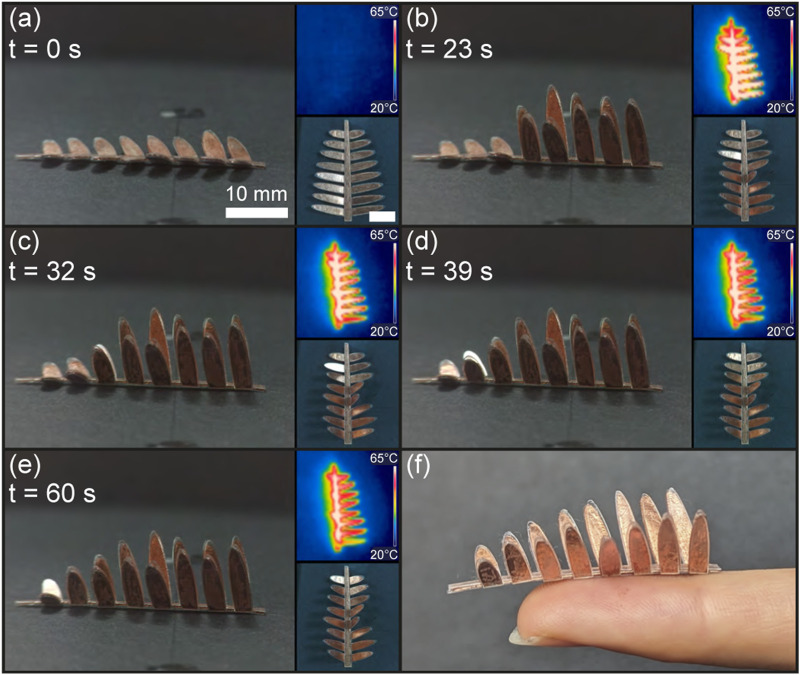
Sequential self-folding of the bio-mimetic Mimosa pudica leaf. The leaf in its flat state **(A)** self-folds its sub-leaves pair 1 to 5 **(B)**, followed by layer 6 **(C)**, 7 **(D)**, and 8 **(E)**, resulting in the fully self-folded Mimosa pudica leaf **(F)**.

This experiment aims to demonstrate sequential folding by varying the heating rate of receivers of different sizes. Inspired by the mimosa pudica, which sequentially closes its leaves upon physical touch, a bio-mimetic self-folding leaf was designed and fabricated to exhibit a sequential folding response to heat. The leaf measures 40 mm in length and 30 mm in width, with 16 sub-leaves ranging in size from 33.8 mm^2^ to 12.1 mm^2^. The sub-leaves are symmetrically arranged along the stem and numbered in pairs from 1 to 8, with each pair having the same surface area of induction to ensure simultaneous folding. The sub-leaves were fabricated using a tri-layer approach, while the stem utilized a blanket configuration to supply heat along its length. The leaf was positioned such that the center of the induction coil was between the third and fourth sub-leaves, providing maximum power to these sub-leaves and decreasing for those farther away (Figure 9A). As shown in Figure 9B and as seen in Supplementary Video S4, sub-leaves pairs 1 to 5 simultaneous folded in 23 s. Depicted in Figures 9C, D, F, sub-leaves 6, 7, and 8 sequentially folded in 32, 39, and 60 s respectively, resulting in the self-folded bio-mimetic Mimosa pudica leaf in Figures 9E, F. Sequential folding using different receiver sizes is therefore validated. This approach to sequential folding may be applied to a wide range of origami patterns by fragmenting the receivers into smaller surfaces, thus reducing the heat rates of individual receivers.

### 3.3 Receiver size and placement for sequential folding

The objective of this experiment is to demonstrate sequential folding of the croissant structure by positioning receivers of varying sizes at different distances from the induction coil center, thereby creating heat rate disparities due to differences in received power.

The croissant consists of five trapezoidal blanket-type receivers arranged in decreasing size, connected at the small base of the trapezoid from largest to smallest. The receiver areas are 136.5, 98.7, 68.8, 46, and 29.2 mm^2^. In the experiment, the unfolded croissant was placed so that the center of mass of the second receiver was aligned with the coil’s central axis. The largest receiver was expected to fold first, followed by the smaller receivers in order of their proximity to the coil center.

**FIGURE 10:**
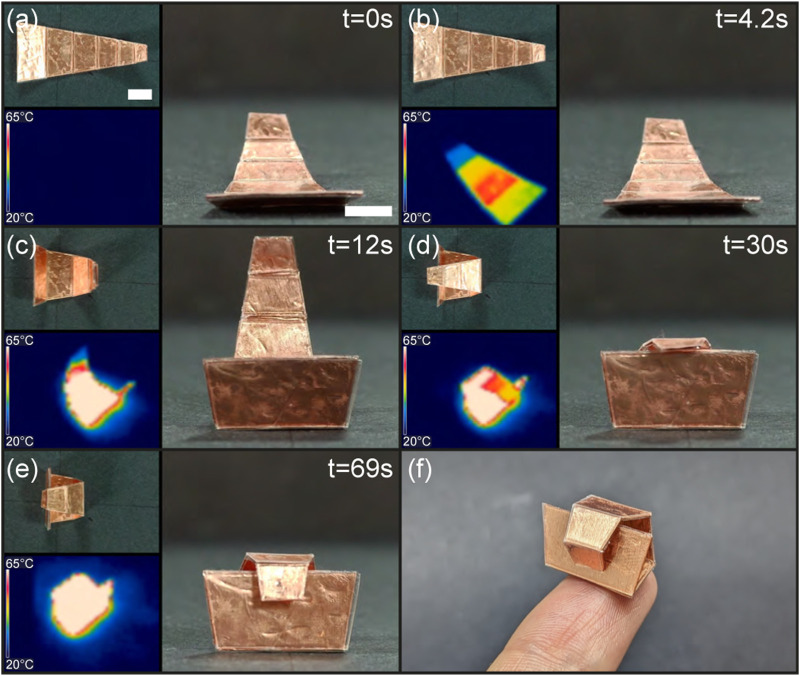
Sequential folding of the croissant by using receivers of different sizes and distances from the center of the coil **(A–E)**, and perspective view of the croissant layer on the tip of an index finger **(F)**. Scale bar is 5 mm.

Figure 10 and Supplementary Video S5 illustrate the folding process, showing the croissant folding sequentially in 70 s into its final rolled shape. In the final stage, the smallest receiver folds around the largest trapezoid base, locking the structure. However, the largest receivers folded simultaneously followed by the predicted order of size of receivers. This is due to insufficient design features, such as holes, to create distinct heat rate variations or magnetic field non-uniformities across the hand-made induction coil, but suggests that the proximity with the center of the coil is the most impacting factor. Additionally, folding in the opposite direction was observed between Figures 10A, B which may be attributed to the thermal dependency of the PVC film’s stiffness and deformation due to the load on the hinges along with the PVC film thermal expansion.

Additionally, the sub-leaves can be seen unfolding as they heat up below 65° C, which can be explained by the influence of thermal expansion that loosen the hinges and unfolds due to the load of the copper receiver. Although, the unfolding is not observed after folding to 90° , the unfolding was observed after fully folding. The large motion amplitude possibly locally delaminates the PVC from the copper receiver around the hinge. This results in a change of rotation axis from the bottom structural layer to the top structural layer reversing the folding with further shrinkage, be it by further activating the PVC or thermal contraction.

### 3.4 Receivers heat capacity for sequential folding

This experiment aims to achieve sequential folding of a box by introducing holes in the receivers to alter their heat capacity.

The cubical box, 12.5 mm wide, has four 11 mm wide blanket-type square flaps with varying hollowness: 0% (white), 30% (blue), 45% (green), and 60% (red). To decorrelate the flaps’ heating rates while maintaining foldability, the central receiver was reduced to an 8 mm square, surrounded by passive material. Opposite receivers were connected with PVC film, coupling the blue/red and green/white receivers to prevent any folding discrepancy due to the PVC film anisotropic shrinkage. Finally, a Dura-Lar out-of-plane angle limiter prevented the folding of the flaps beyond 90° and their collision when over-folding.

**FIGURE 11:**
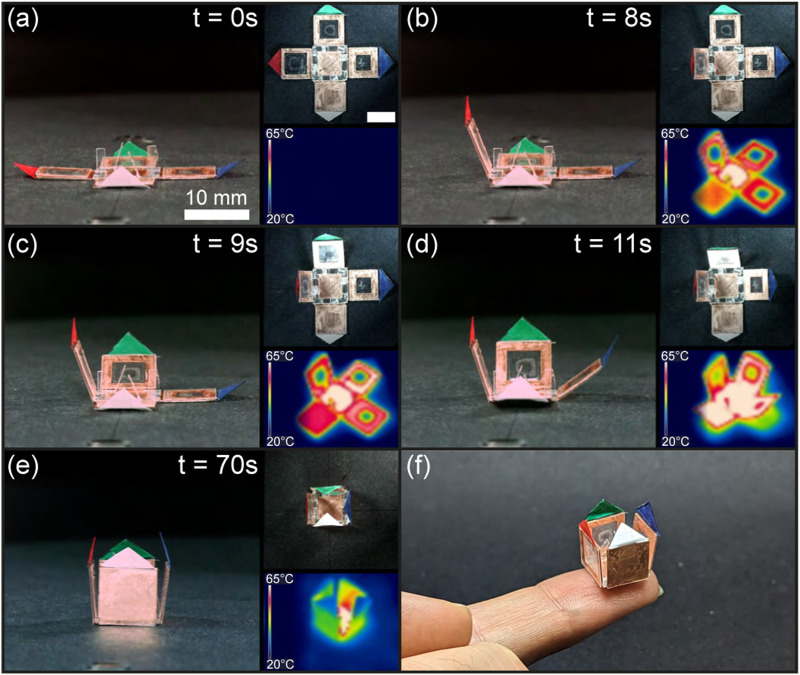
Sequential folding of the box. The structure in its flat state **(A)** folds first its receiver with an 8 mm wide hole (red) **(B)**, followed by the receiver with a 6 mm wide hole (green) **(C)**, the 4 mm wide hole receiver (blue) **(D)**, and the plain receiver (white) until completion of the folding **(E)**. Pictures from another perspective **(F)**.

As a result, when placed at the coil center, the box self-folded in 70 s following the expected sequence. As shown in Figure 11 and Supplementary Video S6, the 60% receiver folded in 8 s, the 45% receiver in 9 s, the 30% receiver in 10 s, and the plain receiver in 11 s.

Unlike the croissant structure, each copper receiver of the box has a distinct hollowness inducing large heat rate discrepancies, and to achieve a clear folding sequence, the impact of reducing the surface area of induction of the central receiver is demonstrated. This method, which can be used at various scales, allows for any receiver to be made hollow and, if necessary, reinforced with rigid material. In addition, this approach can be applied to various structures, such as a 1D self-folding string or a 2D origami pattern.

### 3.5 Self-folding of the overhand knot

To fold an overhand knot, the loop must form first, followed by the helix passing through the loop. This can be achieved by altering the design and size of the copper receivers to self-fold the knot.

The methodology developed in Section 2.1 proposes forming a polygon loop with a strip whose ends do not cross. The loop forms a square with an internal length of 16 mm, covering 676 mm^2^ around the center of the induction coil. Instead of adding a hinge to the first segment of the loop as mentionned in Section 2.1, it was manually bent upward before the experiment to prevent the loop ends from colliding during self-folding. Hollow receivers were used for the loop, placed centrally in the workspace to increase their heat rate. In addition, the length of the knot and in-plane motion introduced frictional torques between the workspace and the knot, causing the knot to displace when folding and the in-plane hinges to stop folding. To limit the influence of friction, two layers of PVC film were introduced to increase the folding torque, and the knot was anchored by gluing receiver $l_{n}$ to the workspace, hence bringing the ends of the loop around the coil center. To create a heat speed discrepancy between the loop and helix, the helix receivers were kept plain, initially positioned further away than the loop from the coil center and with the first helix receiver being smaller to reduce power induced. When unfolded, the overhand knot string measures between 5 and 7 mm wide wide, 135 mm long, with six hinges split equally between the helix and loop. These are equipped with 127μ m thick Dura-Lar to insulate the receivers and limit the folding angle.

**FIGURE 12:**
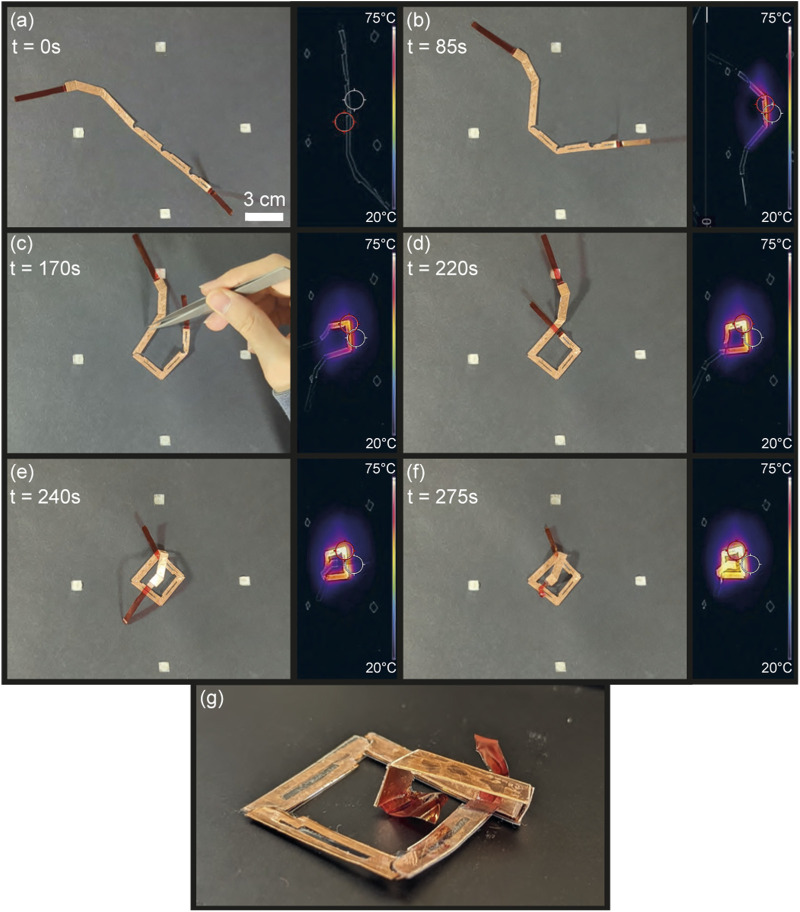
Sequential self-folding of the overhand knot. The knot is first in a flat state **(A)**, folds its loop **(B, C)** before folding the helix parallel to the plane of the loop **(D, E)**, and passes through the loop **(F)** giving the perspective view in **(G)**.

Figure 12, as well as the Supplementary Video S7, demonstrate the self-folding of the overhand knot and its infrared thermal readings. As shown in Figure 12B, the loop heats and folds first in 220 s, with slight manual intervention required to push receivers $l_{1}$ and $l_{n}$ to their 90° target. After this, the rest of the knot folded without assistance. As the loop folded, it brought the helix within the self-folding region as seen in Figure 12C. From 170 s onward, the helix began folding, thereby completing the self-folding overhand knot after 275 s.

The overhand knot self-folding was conducted in four trials, each requiring human intervention to fully fold the loop. Single hinge tests in the workspace’s center showed high success rates for in-plane hinges (Supplementary Figure S2). This implies that the out-of-plane torque induced by the length of the knot causes the in-plane hinge receivers to unhinge, intertwine and lock. In addition, improper insulation placement can cause the folded in-plane hinge receivers to contact, increasing the surface area of induction, sharply raising the temperature, and potentially melting the PVC film. Therefore, further design iterations and trials are needed to improve the in-plane hinge’s resilience to out-of-plane torques and safety. Additionally, to prevent the friction from offsetting the knot placement, the knot was anchored with glue, which restricted the PVC film from receiver $h_{m}$ from fully passing through the loop. In spite of these limitations, the self-folding of the overhand knot was validated following our design methodology.

## 4 Discussion

### 4.1 Comparison with the literature

Here our system working at 150 kHz and 3.2 mT enabled the heating by eddy currents of non-ferromagnetic materials and allowed the folding of origami structures. To date, self-folding is mainly achieved by hysteresis heating of micro- or nano-particles embedded within smart materials. The dispersion of such particles requires careful adjustments to the rheological properties and prevention of agglomeration to maintain the uniformity of the heating. Additionally, the heating requires magnetic fields with amplitudes exceeding 15 mT and frequencies superior to 200 kHz, limiting the penetration of the magnetic field due to the skin effect and reducing the biocompatibility with increased power. To leverage the maximum power output from the induction heating, the system generally works at resonance frequency which, requires a balance between the inductance and capacitance of the system (see equation in Supplementary Section S1). As the induction coil inductance is dependant on its geometry, number of turns, material permeability and wire thickness, increasing the resonance frequency leads to a reduction in size of the induction coil, hence demonstrating a lower workspace surface in comparison to our system. Although our system works at a relatively high frequency to be considered fully biocompatible ( <100 kHz on Non-Ionizing Radiation Protection (2020)), the large number of variables on the origami structures (thermal insulation, hollowness, electric resistance, shape of copper receivers, surface of induction, proximity) allow the heat generation optimization to further reduce the working frequency.

### 4.2 Convection coefficient in self-folding systems

The convection coefficient h significantly influences the folding dynamics of origami structures using induction heating. Although in our study, the value of h is considered constant, in reality h varies with experimental conditions, geometry, flow field, and material properties. It is noteworthy to consider that, under certain conditions, there could be a transition from natural convection to turbulent flow, which strongly alters h and consequently, affects the uniformity of heat distribution and folding dynamic. Additionally, if the folding robot is in motion, the induced airflow could further complicate the heat transfer, requiring dynamic evaluation of h . In regards to the scaling of the copper receivers, different sizes and shapes can also alter h . Thus, a more rigorous approach, potentially involving computational fluid dynamics (CFD) simulations and experimental validations, is required to predict h accurately and ensure reliable folding behavior across various conditions. Moreover, due to the multi-physics nature of the system, optimisation of the folding sequence requires numerical simulation softwares to accurately predict the folding sequence and angles in dynamic environments. Additionally, the system parameters must be tailored specifically for a given application and environment, thus necessitating empirical tests to validate the folding sequence, and assess the limitations of a magnetic induction robot folding within the environment. Examples include distance from the coil, heat losses or collisions with the environment.

### 4.3 Blanket configuration limitation

**FIGURE 13:**
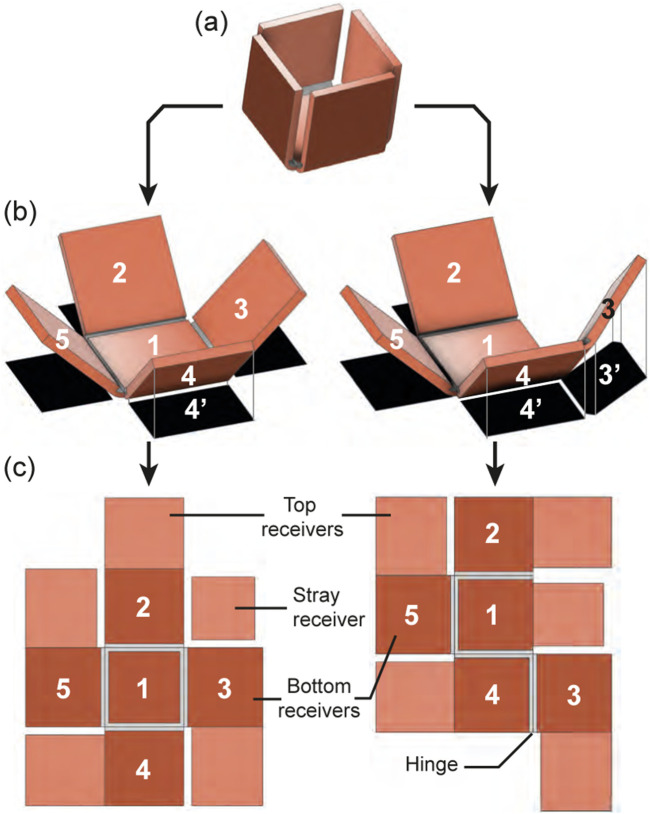
Limitation of the blanket configuration. The target box structure **(A)** has different possible folding pattern **(B)** (with numbered bottom panels in dark orange and top panels in light orange) which gives a folding configuration **(C)** with projected surface area of induction in black.

Fabricating self-folding structures presents challenges, particularly in the precise positioning and alignment of top and bottom receivers and thermal insulators, which leads to incorrect folding angles and sequences. While the blanket configuration helps align the receivers, its applicability is limited to certain patterns. For instance, in the crease pattern of the box in Figure 13A, the central receiver cannot use the blanket method and must be manually positioned. However, modifying the crease pattern in Figure 13B allows all hinges to use the blanket configuration. Although both patterns result in the same box structure, the axis of rotation for the amended receiver 3 depends on its attachment to receiver 4 (Figure 13C). Assuming that the central receiver aligns with the coil center, reduced heat generation from increased distance and double rotation between receivers 4/1 and 3/4 causes receiver 3’s coupling to decrease rapidly. Consequently, receiver 4 folds first, bringing receiver 3 perpendicular to the coil and stopping its heat generation. This highlights the limitations of the blanket configuration and crease pattern design for planar self-folding structures. To circumvent this limitation, a tri-layer configuration at the center can be introduced while adjusting the hollowness of the copper receivers to compensate for the heat rate discrepancy between tri-layer and blanket configurations.

### 4.4 Miniaturization of self-folding structures

Adjusting the design of the receivers and their layer configuration increases heat rates, facilitating the scaling down of structures. For miniaturization, the PVC film can be replaced with another thermo-responsive shrink film with a lower glass transition temperature, such as polyurethane film. Alternatively, the power of the induction coil can be increased, or the environmental temperature elevated. Custom-made bio-compatible polyurethane filaments, shrinking at 40° C, have been demonstrated for self-suturing devices, indicating the potential for induction-based structures to self-fold at lower temperatures and *in vivo* Jing et al. (2016); Lendlein and Langer (2002). However, higher frequency introduces hysteresis heating Razzaq et al. (2007); Deatsch and Evans (2014), requiring a different model Jaafar (2013) than the one developed here. Additionally, thermal insulation becomes less effective at smaller scales, as explained by Fourier’s law of thermal conduction, thus requiring a balance between scale and the power output of the induction coil.

### 4.5 Workspace and up-scaling the size of self-folding structures

The self-folding region is smaller than the induction coil surface because the magnetic field amplitude decreases quadratically with the distance from the coil center. However, this region size can be adjusted by increasing the coil power, frequency, and geometry, or by changing the receivers’ size, design, layer configuration, magnetic materials, and thermal insulation. Increasing the size of the coil to increase the self-folding region is also a viable approach, although the geometry affects the uniformity of the magnetic field along with the resonance frequency, which requires a balance between the inductance L and capacitance C of the magnetic induction system defined as $f_{\mathrm{res}}=\frac{1}{2\pi \sqrt{LC}}$ , hence limiting the resonance frequency for a given geometry. Scaling up structures involves comprising all hinges within their self-folding regions, which can be defined as the area of the coil where a given hinge receives enough heat to fully fold. This leads to self-folding regions per hinge rather than per structure. Aligning these regions with the induction coil center ensures the entire structure will self-fold, either by entering the self-folding regions or, alternatively, by enabling the navigation of the partially folded structure to bring the unfolded sections into their self-folding regions. Consequently, this would increase the success rate of larger self-folding structures and expands the range of self-foldable designs.

### 4.6 Induction coil and safely self-folding region

As mentioned in Section 2.5, overheating of the PVC film generates highly toxic, corrosive white fumes of hydrogen chloride. To prevent the production of these fumes, it is crucial to consider overheating regions in addition to the self-folding region, ensuring that any copper receiver remains below the PVC film’s melting temperature of 136° C. Overheating regions are influenced by the coil power, the distance between the copper receiver and the coil center, the design of the self-folding structure, the materials selected, and the duration of the experiment. Consequently, experiments should be conducted in a well-ventilated area, such as a fume hood, for safety reasons unless both the self-folding and overheating regions are accurately estimated.

## 5 Conclusion

In conclusion, this study presents a novel approach to sequentially self-fold an origami into a target structure through magnetic induction heating. The methodology involving modifications to the receivers, including design, size, configuration of receivers, as well as their placement on the induction coil, was successful for the achievement of sequential folding with the main factor being the distance coil/receiver. This mainly manifested by tuning the heat rate of each receiver highlighting the existence of self-folding regions to properly self-fold structures. The fabrication process of magnetic induction-based self-folding structures was established, and the magneto-thermal model was developed to predict folding sequences based on design parameters of the self-folding structure and magnetic field characteristics. The effectiveness of this approach was validated through thermal and folding speed tests on various structures, including a croissant, a box, and a biomimetic Mimosa pudica leaf. Additionally, the methodology was successfully applied to the self-folding of an overhand knot with minimal assistance at this stage, illustrating its potential for complex structures such as self-locking structures including knots.

This research introduces design recommendations for induction heating-based sequential self-folding, which expand the range of foldable structures as small as 5 mm. In the future, further reducing the working frequency to increase the depth penetration and biocompatibility of the system, while maintaining sufficient heat generation for self-folding, is necessary to introduce induction heating based origami for *in vivo* applications. This includes self-stitching sutures to reduce surgical invasiveness and post-surgery infections, contingent upon the use of biocompatible materials with lower activation temperatures such as polyurethanes. Furthermore, the design parameters for creating folding sequences can be utilized to prevent the unfolding of origami structures or for applications requiring self-locking to secure cargos such as drug delivery or biopsy.

## Data Availability

The raw data supporting the conclusions of this article will be made available by the authors, without undue reservation.
