# Supplementary material for: Remotely actuated programmable self-folding origami strings using magnetic induction heating
Source: Front Robot AI. 2024 Aug 30;11:1443379. doi: 10.3389/frobt.2024.1443379 (PMC11392685; doi:10.3389/frobt.2024.1443379)
Supplement: Supplementary file 2 [file DataSheet1.pdf]

Supplementary Material

1 INDUCTION COIL SETUP

The induction heating system is key to the self-folding of our structures. Its primary output include the magnetic field strength, frequency, and the size of the magnetic field. These parameters are determined by the coil's inductance, the system's electrical capacity, and the current supplied. The inductance depends on the coil geometry, i.e. the thickness of the wires, the number of turns of the coil, its surface area, and its length. The circular coil used in this system consists of seven turns of wire, each made up of 200 strands of magnetic wire (Farnell: ECW0.2) manually wound to span $96\,\mathrm{cm}^2$, with $4\,\mathrm{mm}$-thick layers, resulting in an inductance of $5.47\,\mu\mathrm{H}$. The capacitance C is matched using the equation $f_{res} = \frac{1}{2\pi\sqrt{LC}}$ which defines the resonance frequency, characterising the frequency at which the system's capacitors and coil balance each other to achieve maximum oscillation amplitude of the magnetic field. We selected a resonance frequency of $140\,\mathrm{kHz}$ to self-fold hinges as small as $6 \times 3\,\mathrm{mm}$. Held by two $4\,\mathrm{mm}$-thick acrylic plates, the center of the coil is situated $7\,\mathrm{mm}$ below the workspace, thus providing a magnetic flux density of $3.2\,\mathrm{mT}$.

2 FABRICATION PROCESS

2.1 Out-of-plane self-folding structure

The fabrication process comprises 7 steps. First, the copper sheet is carefully laid on a sticky mattress to avoid introducing air below the copper sheet. The mattress is then fed into the vinyl cutter (Silhouette Cameo 3) and the pattern is cut (Figure S1 (a)). Once cut, the unwanted sections of copper should be peeled off to avoid damaging the structure when removing the copper in later stages. Similarly, the silicone tape is laid and processed (Figure S1 (b)). Pressure is applied to the silicone tape to ensure that air is not trapped between the layers and the tape is properly glued onto the copper (Figure S1 (c)). Thus, the thermal conduction through the silicone tape is more uniform and the protective layer of the silicone glue can be removed without peeling off the glue. The PVC film is then placed onto half of the pattern, cut by the vinyl cutter and the excess material is removed (Figure S1 (d)). Pressure is applied once again and the PVC film side of the pattern (Figure S1 (e)) is folded back onto the other half (Figure S1 (f)). To ensure that all layers are glued properly, pressure in applied once more and the sticky mattress is removed from the vinyl cutter. Finally, the PVC film/copper composite is peeled off and any tracks of glue from the silicone glue or the sticky mattress are dissolved using a cotton bud soaked in with isopropanol (Figure S1 (g)). As a result, there is no remnant of glue around the hinges that could act as a buttress and limit the folding angle.

2.2 In-plane self-folding structure

Figure S2 (a) to (d) illustrates the self-folding of the latest version of the in-plane hinge with a 90° target folding angle. This version of the in-plane hinge started folding after $30\,\mathrm{s}$ and took an average of $100\,\mathrm{s}$ on five samples to fold to 90°. During the initial stages of the folding, the folding angle discrepancy is of $3^\circ \pm 3.8^\circ$, reaches a maximum of $62^\circ \pm 21^\circ$ during the transient stage before stabilizing to $88^\circ \pm 8.5^\circ$ in the final stages of the folding as shown in Figure S2 (e). Overall, out of the 5 samples, only one failed to fold beyond 80° while the other samples folded between 90° and 100°.

Although the fabrication is similar to that of out-of-plane hinges, some adjustments are required to maximise the performance of the in-plane hinges. As can be seen in Figure S2 (a) to (d), the receivers directly above the bridge are covered with Dura-lar. During the fabrication process depicted in Figure S1 (c), (e) and (h), pressure is applied on the whole surface to prevent delamination and improve thermal contact. The main function of the Dura-lar, other than insulation, is to act as a guide during the folding to prevent out-of-plane motion, to smooth the folding, and prevent the opposite receivers to tuck within one another. Even though these panels are thick enough to function as rigid guides, the material's flexibility is high enough above 65° C to occasionally bend, causing the Dura-lar to tuck between one another. For this purpose, two layers of Dura-lar were placed onto each other to reduce the bending. Nonetheless, these two Dura-lar layers requires pressure to be applied onto them while keeping the PVC film bridge free to shrink to ensure their glueing. Hence, a $50\,\mu$m thick spacer was slid between the receiver and the PVC film before applying the pressure to avoid pinching it. Although the spacing reduces the efficiency of the heat transfer to trigger the shrinkage of the PVC film bridge, this reduces the friction within the hinge and prevent premature stoppage during the folding. Lastly, to prevent the blanket to open during the folding, the top and bottom Dura-lar panels extend beyond the width of the receivers and connects to each other thanks to a thin Dura-lar spacer. Additionally, this width extension lowers the center of rotation of the hinge during the initial stage of the folding to increase the folding torque.

Despite the modifications to improve the folding success rate of the in-plane hinges, the reliability of the hinges regarding their folding accuracy and smoothness of the folding is low. The main limitation being the flexibility of the Dura-Lar layer (due to the material mechanical properties and thickness), a stiffer material able to withstand temperatures reaching $100°$C is required.

3 MODELLING OF THE PVC FILM SHRINKAGE EXTENT AND INDUCTION HEATING

3.1 Folding and shrinkage extent

Depending on the design of the hinge, the extent of PVC film required to shrink differs to self-fold to the targeted angle. Using the equation developed by **?** calculating the folding angle for a given gap width w_g, the shrinkage extent of PVC film required to self-fold to the target angle can be estimated. Here for an out-of-plane hinge based on tri-layer configuration, we found that $10,38\,\%$ of shrinking is required regardless of the gap width of the hinge. Considering that the PVC film shrinks to a maximum of $40\,\%$, the curves in Figure S3 implies that $25\,\%$ of the PVC film within the hinge should be activated to fold to the target angle. From this conclusion, the model requirement can be adjusted to a specific design of hinge.

This same principle can be applied to the in-plane hinge. With the current design, the initial bridge length di_{bridge} is 12.525 mm, while the final length is $df_{bridge} = 7.569$ mm, thus leading to a required shrinkage of the bridge of 37%, and implying a requirement of near 100% of the PVC film shrinkage to fold the in-plane hinge to $90°$. This suggests that any mis-fabrication can significantly impact the performance of the hinge, although 3 hinges were seen to fold past the $90°$ target.

3.2 Induction heating model

Passing through the various layers of our structures, the heat is conducted towards the PVC film or the environment. From the power induced in the receiver by the induction coil using the equations detailed in the Section 2.5 , the model calculates the temperature of the Dura-lar. The Simulink model focuses on the heat transfers within our structures and consists of conduction, radiation and convection transfer blocks positioned between the environment and the materials making our structure. These blocks hold the physical and thermal characteristics of the materials whose values are detailed in Figure S5. Along with these values, the convection coefficient h was set to $46\,W/(^{\circ}Km^2)$ for the experiment of the receiver surface design (Figure 6 (c)) , and $20\,W/(^{\circ}Km^2)$ for the tilted experiment Figure 6 (d)

3.3 Figures

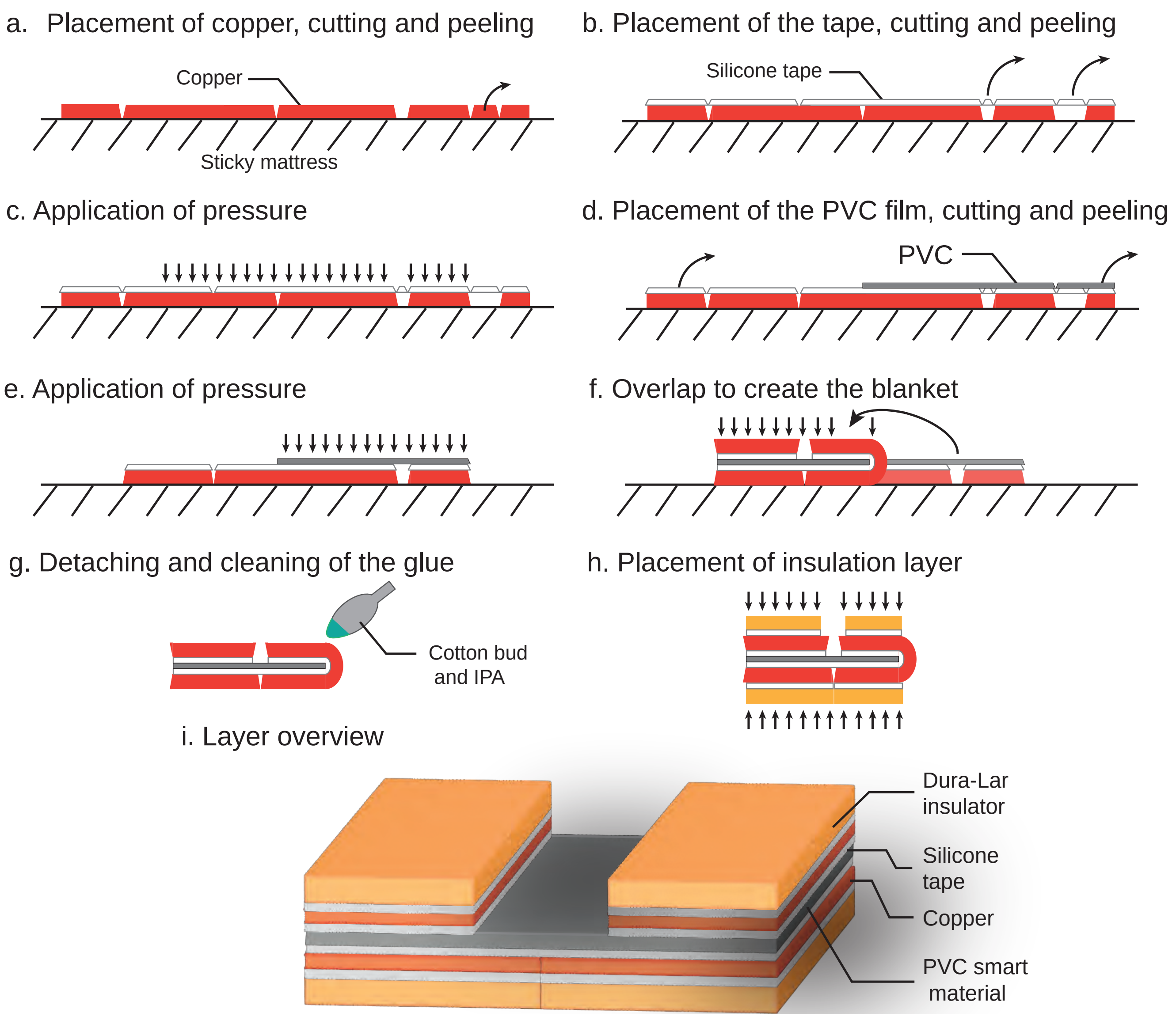

Figure S1. Manufacturing process of the PVC film/Copper blanket tri-layer. The copper sheet is placed onto a sticky mattress and cut (a), then the double sided silicone tape is layed onto the cutted pattern, and pressure is applied to ensure correct glueing (c). The PVC film is placed and unused pieces are removed (d), before pressure is applied again (e). The PVC film is tucked underneath the copper blanket (f), and the structure is detached and cleaned using a iso-propanol (g). Using silicone tape, the Dura-lar is glued to the copper receivers and pressure is applied to ensure proper contact between each layer (h). Overview of the layers on a hinge (i).

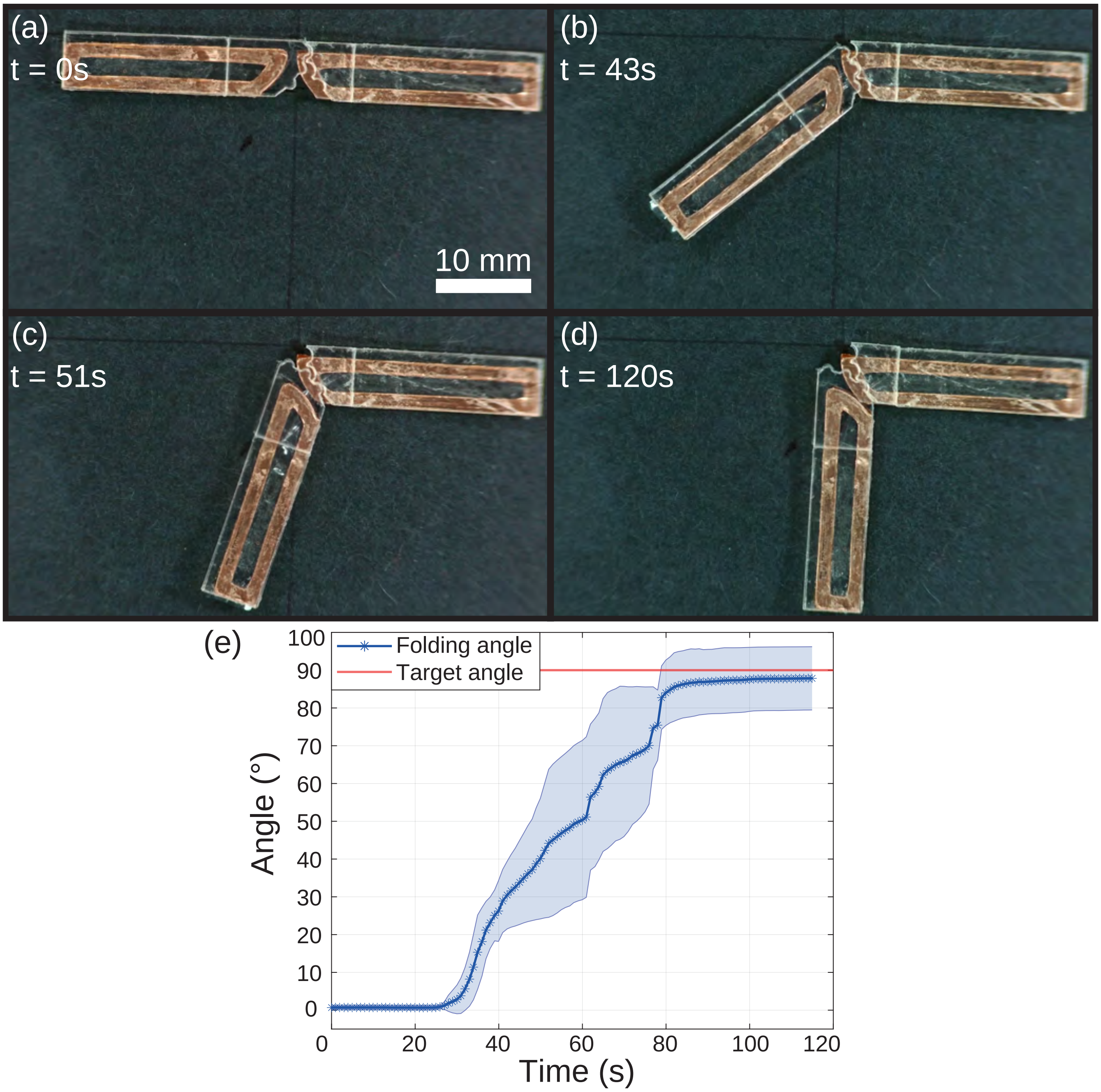

Figure S2. In-plane hinge in its straight configuration (a), and self-folding progressively to $40°$ (b), $80°$ (c) and reaching its target angle: $90°$ (d). Average folding angle for five samples (e).

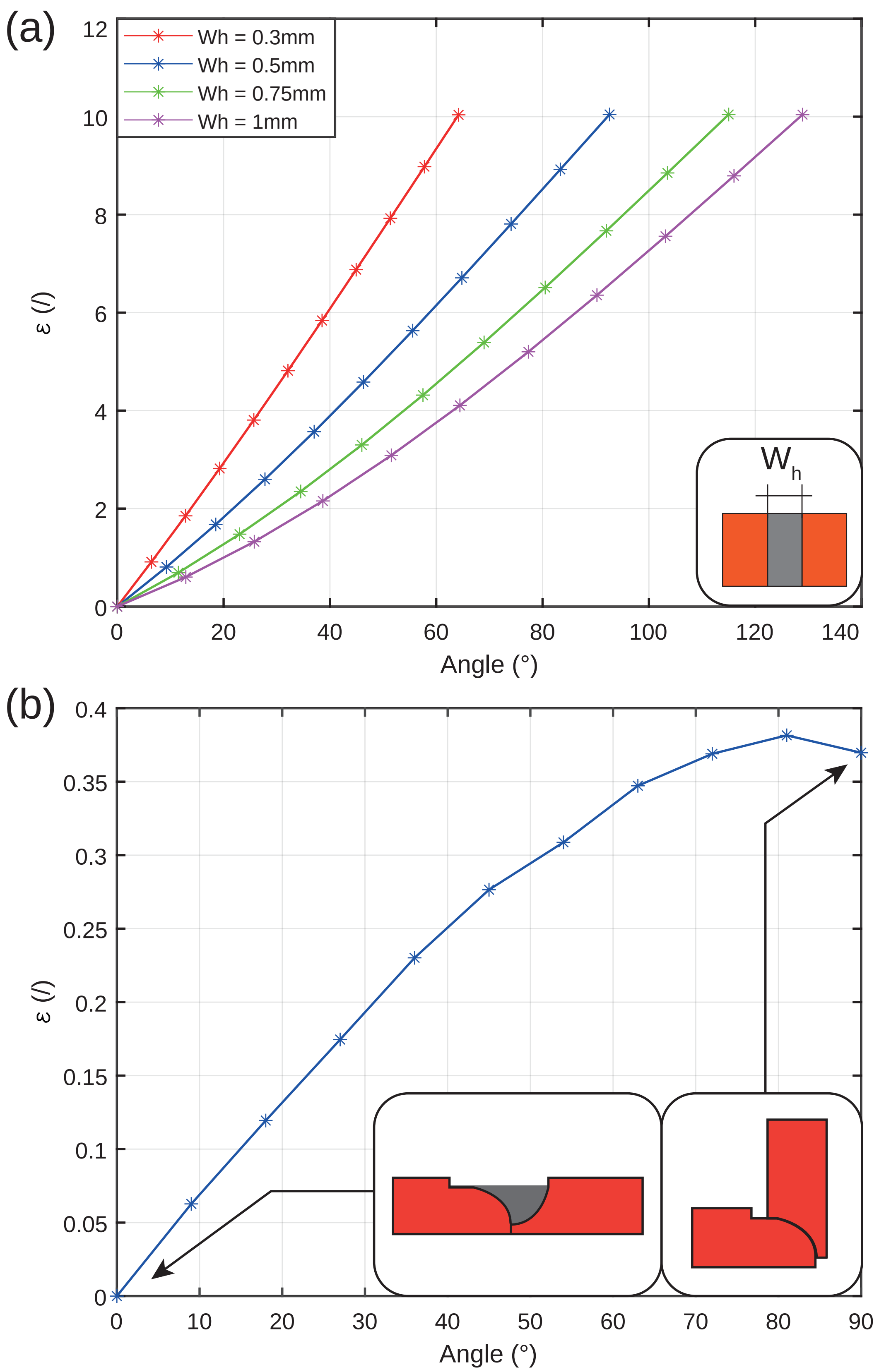

Figure S3. Extent of PVC film shrinkage required to fold to the angle θ for out-of-plane hinges with different gap width (a) and in-plane hinges (b).

(a)

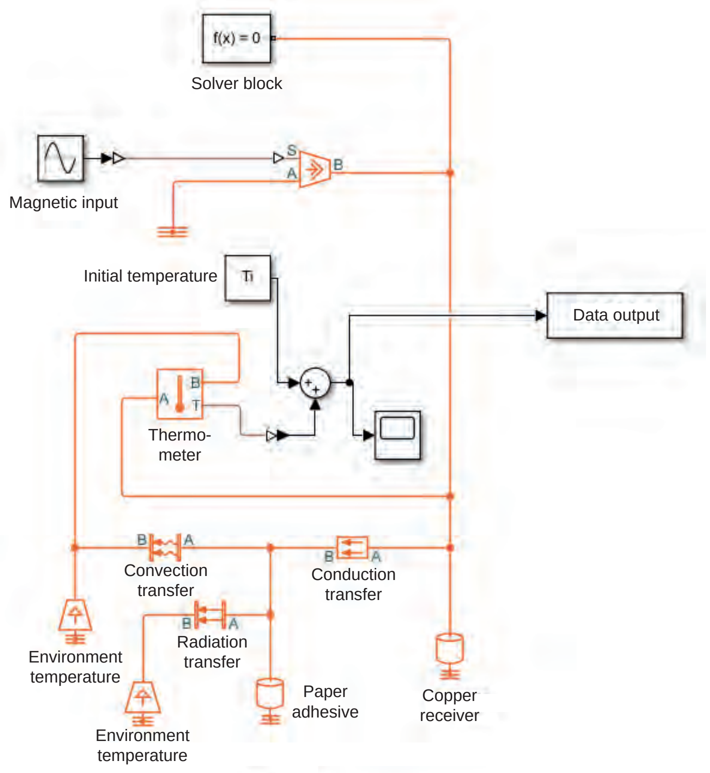

(b)

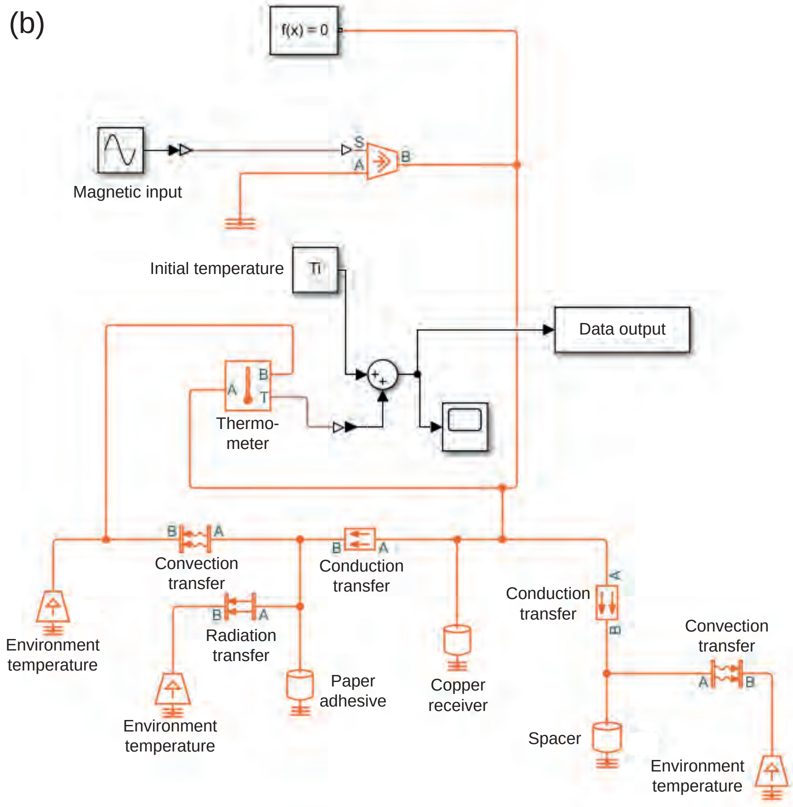

Figure S4. Simulink block diagram for the modelling of the design of the receivers (a) and the inclined receivers (b).

Materials characteristics							
	Thickness (mm)	Density (kg/m^3)	Resistivity (Ω/m)	Permeability (H/m)	Emissivity (/)	Thermal conductivity (W/m°K)	Heat capacity (J/kg°K)
Copper	0.05	8960	1.77×10^{-8}	1	0.2	0.39	385
Silicone tape	0.025	1200	/	/	/	0.2	1050
Dura-lar	0.127	1380	/	/	0.85	0.05	1030
PVC	0.024	1380	/	/	/	0.19	880
Clear resin	5 - 6	1210	/	/	/	0.45	500
Paper adhesive	0.2	1200	/	/	0.9	0.03	1400

Figure S5. Thermal and magnetic characteristics of the materials used for our self-folding structures.
